# Additive Manufacturing of Polyolefins

**DOI:** 10.3390/polym14235147

**Published:** 2022-11-26

**Authors:** Fotis Christakopoulos, Paul M. H. van Heugten, Theo A. Tervoort

**Affiliations:** 1 ETH Zürich, Department of Materials, Vladimir-Prelog-Weg 5, 8093 Zurich, Switzerland; 2 Processing and Performance of Materials, Department of Mechanical Engineering, Eindhoven University of Technology, P.O. Box 513, 5600 MB Eindhoven, The Netherlands

**Keywords:** additive manufacturing, polyolefins, polyethylene, polypropylene, polymer processing, 3D printing, selective laser sintering, fused filament fabrication

## Abstract

Polyolefins are semi-crystalline thermoplastic polymers known for their good mechanical properties, low production cost, and chemical resistance. They are amongst the most commonly used plastics, and many polyolefin grades are regarded as engineering polymers. The two main additive manufacturing techniques that can be used to fabricate 3D-printed parts are fused filament fabrication and selective laser sintering. Polyolefins, like polypropylene and polyethylene, can, in principle, be processed with both these techniques. However, the semi-crystalline nature of polyolefins adds complexity to the use of additive manufacturing methods compared to amorphous polymers. First, the crystallization process results in severe shrinkage upon cooling, while the processing temperature and cooling rate affect the mechanical properties and mesoscopic structure of the fabricated parts. In addition, for ultra-high-molecular weight polyolefins, limited chain diffusion is a major obstacle to achieving proper adhesion between adjunct layers. Finally, polyolefins are typically apolar polymers, which reduces the adhesion of the 3D-printed part to the substrate. Notwithstanding these difficulties, it is clear that the successful processing of polyolefins via additive manufacturing techniques would enable the fabrication of high-end engineering products with enormous design flexibility. In addition, additive manufacturing could be utilized for the increased recycling of plastics. This manuscript reviews the work that has been conducted in developing experimental protocols for the additive manufacturing of polyolefins, presenting a comparison between the different approaches with a focus on the use of polyethylene and polypropylene grades. This review is concluded with an outlook for future research to overcome the current challenges that impede the addition of polyolefins to the standard palette of materials processed through additive manufacturing.

## 1. Introduction

One of the largest global market shares of materials is occupied by plastics due to the attractive compromise these materials offer between their properties and ease of processing, leading to a broad range of applications [1,2]. Polyolefins, also called polyalkenes, are described by the general chemical formula $CH_{2}CHR$, with *R* being an alkyl group and can be found in many industrial and consumer applications [3]. Polypropylene and polyethylene grades are the most common polyolefins by market share and represent around 40% of the total plastic consumption, mostly in areas such as food packaging, the automotive industry, and the medical sector [4,5]. However, polyolefins are also used for high-end engineering applications, for instance, during the construction of offshore wind turbines [6] and in ballistic applications [7,8]. The reason that polyolefins, such as polyethylene and polypropylene, are widely used is due to the relatively easy processing and low cost combined with good mechanical properties and superb chemical resistance [9].

Polyolefins are commonly processed through formative manufacturing techniques, such as injection molding, blow molding, compression molding, or melt extrusion, and subtractive manufacturing techniques, for example, compression molding and extrusion, followed by machining of the molded plate or extruded filament to the desired geometry. The control over several processing parameters of these techniques enables the fabrication of products with the desired mechanical and aesthetic properties. These consist of the temperature and residence time that are adjustable to allow for complete re-entanglement, leading to the desired mechanical properties, the control over the pressure and the cooling rate that enables the utilization of the crystallization kinetics toward the tuning of the mechanical properties and the mesoscopic morphology [10,11]. Moreover, the aforementioned manufacturing techniques are usually characterized by high productivity [11].

In addition, physical properties, such as molecular composition, molecular weight, entanglement density, linearity, and tacticity, are important to the melt viscosity and the developed morphology [12,13]. There is a deep understanding of these well-established processing techniques, and industrial infrastructure is in place that is capable of processing large amounts of polyolefins into standard products. Nonetheless, the improvement and prediction of polyolefin properties, such as an enhanced life of the fabricated specimen to meet the requirements for specific applications, is still an active field of research [14,15].

Additive manufacturing, also called 3D printing, is the process of building up a three-dimensional (3D) object from computer-aided-design (CAD) models through a layer-by-layer fabrication process [16,17,18]. Here, the CAD design is converted to a stereo-lithography file (stl-file) to describe only the surface geometry of the 3D design. The software of the 3D printer converts the stl-file into two-dimensional layers stacked on top of each other [19,20]. Complex geometries can be built in this way while using a variety of materials, depending on the method employed. The successive deposition of layers is a relatively slow process and is, therefore, still limited in terms of quantity. Some benefits of using additive manufacturing techniques are the increase in design freedom and flexibility and the reduction in material waste and production cost [21]. A notable drawback is the reduced mechanical performance of 3D-printed polymer objects, which has been, until now, generally lower than conventional processing methods. This decrease in properties is mostly due to reduced adhesion upon stacking of the layers [22,23]. Reduced consolidation can stem from characteristics of the feedstock material or from defects arising from the fabrication process, such as porosity due to incomplete fusion, keyhole porosity, residual stresses, and warping, amongst others [24,25,26,27,28,29,30]. In the following section, the different additive manufacturing approaches are presented, together with process-specific challenges and limitations. Currently, additive manufacturing is utilized for the processing of polymers, metals, and ceramics [18,31,32,33,34,35,36,37]. Regarding polymers, the focus of the present review is several amorphous and semi-crystalline polymers that are readily processed through 3D printing, such as acrylonitrile butadiene styrene (ABS), polylactic acid (PLA), polycaprolactone (PCL), polyether-ether-ketone (PEEK), Polyamide (PA12), polyethylene terephthalate (PETG), polyvinyl butyral (PVB), etc. However, most are regarded as commodity plastics. Due to the aforementioned limitations, additive manufacturing of polymers has been utilized mainly for rapid prototyping and case-specific applications, for example, prosthetics for medical applications or custom engineering components [35,38,39]. However, there is a recent tendency for additive manufacturing to move from a method for rapid prototyping toward the manufacturing of functional parts aimed at high-end engineering applications. Toward that end, the addition of polyolefins, such as polypropylene and polyethylene, to the palette of standard materials used for 3D printing will be highly beneficial, as they are readily used for high-end applications due to their superior mechanical properties and chemical resistance.

In the present review, the two main methods used for the additive manufacturing of polyolefins are presented, and the work performed on additive manufacturing for their application to polyolefins is explored, with a focus on polypropylene and polyethylene grades. The properties achieved through different processing techniques are compared, and the current limitations of additive manufacturing approaches are discussed.

## 2. Additive Manufacturing Techniques Used for Polymers

### 2.1. Fused Filament Fabrication

**Figure 1:**
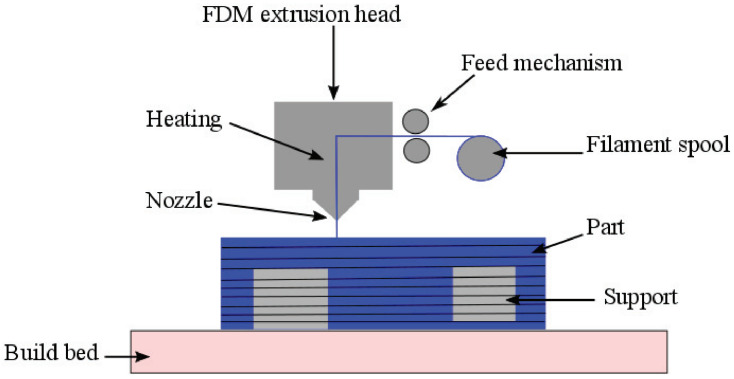
Schematic depiction of the fused filament fabrication process. The filament enters the extrusion head, which heats up the filament coming from the spool, after which the filament is extruded from the nozzle. The material is deposited onto the previously deposited layer to fabricate the desired object. Support material can also be extruded, which is removed afterward, to allow for a higher degree of complexity in the 3D-printed objects. Reproduced with permission [41], Copyright 2017, Elsevier.

One of the most widely used additive manufacturing methods is fused filament fabrication (FFF), which is also known as fused deposition modeling^TM^ (FDM^TM^). In this process, a filament is extruded continuously out of a nozzle, and layer-by-layer, the material is deposited by simultaneously moving the extrusion head. The thermoplastic material is fed from a spool to a nozzle, where it is heated above its melting temperature before being discharged and deposited, as can be seen schematically in Figure 1 [40,41].

Adhesion between two adjacent layers is typically achieved through the diffusion of the covalent-bonded macromolecular chains across the weld lines upon cooling of the material [31,42,43]. At the welding interface, the strength is, therefore, dependent on the extent of chain diffusion and the formation of entanglements or co-crystallization across the interface upon cooling [44,45,46]. Due to the filament extrusion, the polymer chains can be oriented, resulting in anisotropic mechanical behavior at the interface. This often results in decreased adhesion between the layers, reducing the mechanical properties of 3D-printed articles, depending on the orientation that follows the print direction [42]. However, using liquid-crystalline polymers, flow-induced orientation during fused filament fabrication can also be used to produce anisotropic objects with favorable enhanced strength and stiffness [47].

As mentioned before, molecular diffusion is an essential mechanism for achieving good adhesion upon joining polymer surfaces. In the case of FFF, the welding of two polymer surfaces is a complex process involving many molecular and process parameters [48]. The driving force of diffusion is thermal energy [49]. The interfacial strength is thus dependent on the thermal history of the filament, and so is the increase in adhesive fracture energy [50]. The buildup of interfacial strength and adhesive fracture toughness is restricted due to the limited amount of chain diffusion that can occur during printing [48]. This is because, upon deposition of a molten filament, there is only a short time window in which the material on both sides of the resulting new weld line remains in the low-viscous melt. Soon after deposition, the molecular mobility of the polymer chains is reduced upon approaching the glass transition or crystallization temperature during cooling [45,51].

McIlroy and Olmsted [45] report that amorphous thermoplastic materials along the welding interface have a notable fraction of disentangled polymer chains, leading to a reduction in tensile strength. However, in the case of semi-crystalline materials, such as polypropylene and polyethylene, the disentangled and oriented material along the interface does not necessarily lead to reduced properties. Disentangled and oriented chains are indicative of flow-induced crystallization that occurs if macromolecular relaxation is slow compared to chain orientation due to the shear rate upon flowing through the nozzle [52,53]. Flow-induced crystallization causes the formation of oriented crystals, which hinders the formation of entanglements across the interface after the deposition of a new layer [53]. To achieve adhesion between the deposited layers, re-entanglement and co-crystallization have to occur across the interface to improve the mechanical properties. In this case, diffusion across the interface is crucial, which occurs relatively slowly, but is also dependent on temperature, where the thermal energy acts as a driving force and time, to allow for the reptation of the macromolecular chains [54].

The resolution that can be obtained through FFF is in the range of 0.05 to 0.5 mm, depending on the material and the parameters used. Therefore, 3D-printed parts exhibit a surface roughness that mainly originates from stacking the layers next and onto each other. This stacking process causes a stepping effect on the layers, meaning that there is a small inclination between two adjacent layers [55]. The surface roughness is thus dependent on printing parameters, such as the layer height, extrusion speed, wall thickness, printing temperature, and deposition path of the filament, among other things. Of higher importance are the layer height and wall thickness, where a decrease in layer height results in a smoother surface [56].

Producing engineering objects with FFF is limited in several ways, with the already mentioned reduced adhesion between deposited filaments being the main reason for the often observed decrease in mechanical properties. There are also other problems that can occur, such as warpage, which is an unwanted twisting of the 3D-printed object that results from shrinkage upon cooling. For amorphous polymers, warpage is less of a problem than for semicrystalline materials, as the amount of shrinkage is only due to the ever-present thermal contraction upon cooling [57].

However, for semi-crystalline polymers, crystallization results in an extra amount of shrinkage due to the higher density of the crystalline phase compared to its amorphous counterpart. The extra shrinking increases the internal shear stresses between two adjacent layers, which promotes warpage [30,58].

An important factor in starting a 3D printing process is the adhesion of the first layer to the print surface. Most commercial FFF printers suggest the use of nonstick coatings, such as polytetrafluoroethylene (PTFE), for easy removal of the printed specimen; however, this could be problematic for the correct deposition of the first layer for certain polymers, especially for apolar polyolefins, as examined in the present review. In the case that the adhesion to the print surface is poor, the first layer may move. As a result, the following layer is deposited not on top of the previous layer. The 3D-printed part will not come out as desired, and the process is thus interrupted. Adhesion of the deposited layer to the surface bed can be improved by using a higher surface temperature. In the work of Spoerk et al. [59], it is shown that a temperature slightly above the glass transition temperature of the printed material results in better adhesion of the deposited layer to the bed surface since the surface tension in the filament is reduced, leading to a larger contact area [60]. Another approach is to coat the printing surface with the same polymer that is used for printing, which would result in superior adhesion of the first printed layer, but, in turn, would render the removal of the printed object more challenging.

### 2.2. Selective Laser Sintering

**Figure 2:**
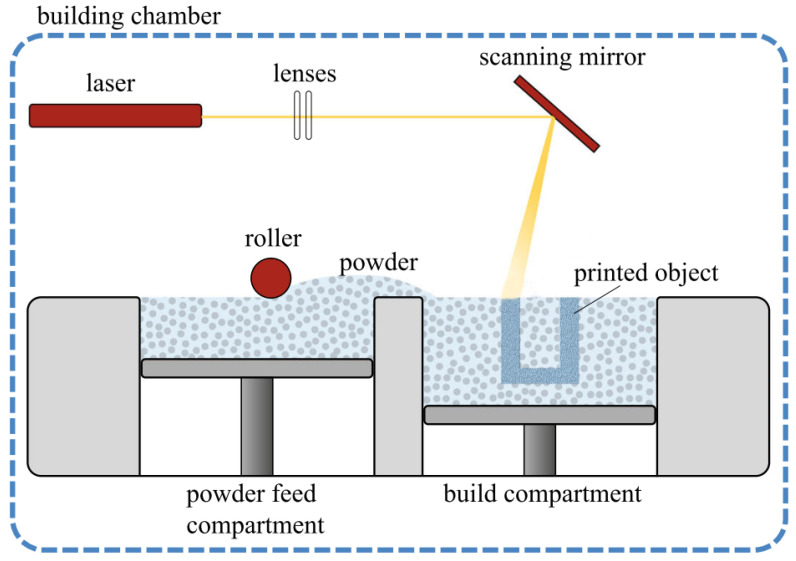
Schematic depiction of the laser sintering process inside the building chamber of the 3D printer. Here, the powder feed compartment holds the polymeric powder, and the build compartment is where the deposition of a new layer of powder and the laser scanning and sintering of a new layer of the printed object takes place. The laser passes through a convex lens onto a scanning mirror, which can rotate to move the laser spot along the surface of the deposited layer. Reproduced with permission [19], Copyright 2020, Springer Nature.

Another additive manufacturing technique is selective laser sintering (SLS). Through SLS, material in the form of a powder is consolidated by the use of heat coming from a laser. In Figure 2, a schematic representation of the SLS printing process is depicted. To form a new layer, a roller or blade first-of-all deposits a new layer of powder at the build compartment from the powder feed compartment. A laser then scans over this newly deposited layer of powder and selectively melts a small amount of powder by locally heating it [16,61,62]. After the layer is built, the build compartment moves down a set increment, and the powder feed compartment moves up. The process of depositing powder and scanning the surface layer with the laser is then repeated for the deposition of each subsequent layer. After the entire 3D design is built, the material is cooled down and subsequently removed from the powder bed [63]. Post-processing of the extracted parts includes removing excessive powder by using, for instance, pressurized air and optionally polishing the surface for aesthetic purposes, as this provides a smooth look.

In an SLS printer, the temperature of both the building chamber and the powder surface is controlled. Before the SLS process starts, a gradual preheating of the powder bed is needed to minimize shrinkage of the printed part and prevent thermal gradients in the powder feed compartment [31]. The powder bed temperature is set to the highest temperature that does not result in consolidation of the powder. The laser is used to deliver the least amount of energy required to consolidate the scanned powder. The temperature is chosen to be as high as possible to prevent thermal gradients between the sintered material and surrounding support material. Further, the temperature of the bed diminishes the effect of the thermal expansion of the powder that is scanned by the laser. When the temperature of the bed is set too low, curling, e.g., warpage, of the scanned layer can occur [63].

During the consolidation of the polymeric powder through SLS, the mechanism to adhere the particles to each other is thought to be based only on the partial melting of the powder particles [64]. The fusion of the particles occurs on very short timescales in the order of seconds, which considerably limits the formation of entanglements, which follows the well-known reptation mechanism. Here, as the temperature of the bed is lower than the melting temperature of the polymeric material, diffusion is restrained by the molecular arrest of the polymer chains [65].

A phenomenon that can arise is the so-called “orange peel”, which is a rippled texture that forms on the surface of 3D-printed parts. The surface texture evolves from the solidstate condensation of unsintered particles [32]. “Orange peel” can occur after recycling the powder since the exposure of the powder to heat leads to thermal degradation [66]. Eventually, the uneven texture also leads to a decrease in the mechanical properties of the fabricated object [31,64]. A different distortion that can occur is warpage. For semi-crystalline polymers processed through SLS, curling of the 3D-printed parts starts to develop upon crystallization [67]. Curling of the sintered layers can thus happen during the printing process, causing geometrical distortions of the sintered part that can result in the breaking off of the printing process when the deformation in the out-of-plane direction is larger than the layer height [68]. Consolidation due to crystallization gives rise to a decrease in the volume of 3D-printed parts. Similarly to FFF, warpage is also present in SLS, with the effect being more prominent for semi-crystalline polymers compared to amorphous polymers [57].

Generally, good adhesion is the limiting factor in generating good mechanical properties. In SLS, both the adhesion between the particles of one layer and between two adjacent layers are of importance [69]. In addition, to enable good adhesion, the spreading of the powder has to be conducted in a way that results in a dense and evenly spread powder bed. For that, the flowability of the powder is important, which, in turn, is dependent mainly on the shape of the particles, its bulk density, the particle size distribution, the presence of agglomerates, and the bed temperature [33,70]. In the work of Schmid et al. [71], it is shown that using the round-robin test, an analytical evaluation of the flowability of a powder can be performed. Here, the Hausner ratio, defined as the ratio between the tap and bulk density, indicates the flowability, where a ratio that is below 1.25 is regarded as a suitable powder for SLS printing based on the extrinsic properties of the material.

Sintered samples are known for their porous morphology, which is influenced by the bulk density of the powder. An increased density would result in a denser final structure, but this would also influence the flowability of the powder [70]. The strength and resolution of the 3D-printed parts are, however, not only influenced by the density but also by the laser speed. The laser speed influences the energy input, which affects the amount of shrinkage, and can also change the amount of time the powder particles stay in the melt [64].

**Figure 3:**
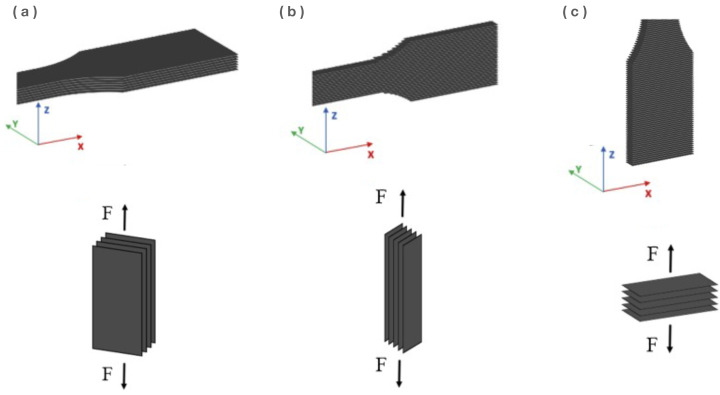
The printing direction of the sintered layers, where the layers of a 3D-printed tensile bar are oriented in (**a**) the *x*-direction, (**b**) the *y*-direction, and (**c**) the *z*-direction. *F* corresponds to the loading direction. Reproduced with permission [72], Copyright 2021, Elsevier.

The direction in which the layers are sintered causes a certain anisotropy in the fabricated object. The building direction can be chosen depending on whether the adhesion between two adjacent layers or the fusion between particles within one sintered layer is more important for the respective application [72]. It is claimed in the work of Calignano et al. [72] that the sintering direction, as depicted in Figure 3, gives rise to a difference in tensile properties and a different distribution of the pore diameters within the 3D-printed specimen. A specimen that is built in the *z*-direction is found to have the lowest mechanical properties [73]. When the specimen is fabricated in the *z*-direction, the tensile properties are only dependent on the adhesion between two adjoining layers that develop through inter-diffusion between the already sintered layer and the newly scanned layer. In comparison, when printing in the *x*- and *y*-directions, the adhesion is dependent on interdiffusion within one layer in one direction.

## 3. Approaches Used for the Additive Manufacturing of Polyolefins

### 3.1. Polypropylene

One of the most commonly used polymers is isotactic polypropylene (iPP) [74]. In additive manufacturing, iPP can be used in both FFF and SLS. Polypropylene is a polymorphic material in which the mechanical properties of the final product can be tuned through the temperature and cooling rate during processing. This is due to the different crystal morphologies that iPP can form, which, in turn, have a very different mechanical response [75]. The best-known polymorphs are monoclinic α-phase, trigonal β-phase, orthorhombic γ-phase, and mesophase (smectic form) [76,77,78,79,80,81].

**Figure 4:**
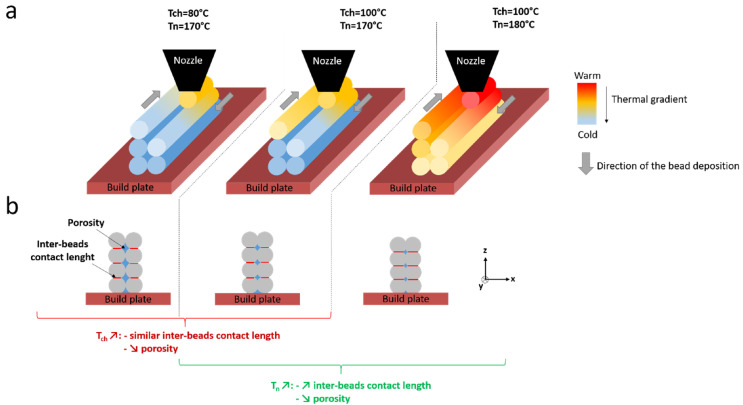
A schematic outline of the influence the chamber temperature $T_{ch}$ and nozzle temperature $T_{n}$ have on the consolidation of deposited iPP filaments, i.e., beads, through FFF (**a**) along the deposited filament and (**b**) along the cross-section in the ZX-plane. Here, a higher chamber temperature lowers the porosity, described as the gap between deposited filaments, and a higher nozzle temperature increases the length between two deposited filaments and decreases the porosity. Reproduced with permission [83], Copyright 2021, Elsevier.

There are reports in the literature of iPP being processed via FFF, with several advances in the past few years [82,83,84]. It should be noted that several commercial filaments for FFF are branded as iPP, whereas these are usually copolymers with different additives. In the work of Silva et al. [82], the mechanical properties of FFF-printed samples come within 70–80% of parts processed through injection molding. The two main difficulties when using iPP for FFF are achieving good mechanical properties and minimizing the effect of warpage stemming from the semi-crystalline nature of iPP. The adhesion between two layers and the porosity of FFF-printed parts is dependent, among others, on printing parameters such as the temperature of the nozzle and bed chamber, as can be schematically seen in Figure 4 [83]. An increase in the temperature of the nozzle has been reported to result in products with higher values of adhesive fracture toughness [28,83]. The melt viscosity decreases at higher temperatures, thus promoting the contact between the newly deposited and the previous layer. Regarding the effect of the temperature of the bed chamber, a lower value increases the undercooling and so the cooling rate, which results in a larger temperature difference between the newly deposited layer and the previous one. This restricts the formation of entanglements and co-crystallization across the interface and, thus, obstructs the consolidation of the two layers. Further, the porosity of FFF-fabricated samples is affected by the bed temperature, with a higher temperature resulting in decreased porosity. From these considerations, it follows that for a semi-crystalline material like iPP, the bed temperature has to be set close to its melting temperature [83].

According to Lotz et al. [77], the most common morphology found in iPP is the α-configuration. This was also found by van Erp et al. [85], who investigated the crystallization kinetics of iPP homopolymer upon constant cooling as a function of pressure and shear flow conditions. They could distinguish three regimes: quiescent crystallization, flow-enhanced point nucleation, and flow-induced crystallization of oriented structures, resulting in morphologies ranging from spherulitic to oriented shish-kebabs. For commercial grades of iPP at low pressures and moderate shear rates, conditions that are typically relevant for both SLS and FFF, crystallization proceeds toward spherulitic or weakly oriented row structures. In this case, the α-phase was found to be the most common, whereas the β-phase only occurs when flow gradients are present. In this study, crystallization of the γ-phase only took place at elevated pressures. Varga [76] stated that the formation of the β-phase of iPP is also promoted by high undercooling, crystallization in a temperature gradient, and, most efficiently, by the use of special β-nucleating agents. Wang et al. [84] showed that, in FFF, the formation of β-crystals occurs, with several explanations being given for its formation. Namely, the favorable temperature of 130°C, at which the bed temperature is set, is beneficial for the formation of β-phase and the re-crystallization of α- to β-phase [86,87]. β-phase can only form on aligned α-phase or by means of nucleating agents. The formation of an aligned α-phase is caused by shear flow at the extrusion nozzle [53,88]. Due to the shear flow, the polymer chains are stretched and oriented, which gives rise to flow-induced nucleation [89,90]. The temperatures of the nozzle and the chamber, together with the extruding velocity of the thermoplastic material, thus have a great influence on the crystallization kinetics and the resulting morphology. A benefit of using FFF over SLS with iPP is the possibility of using a reinforced filament as feedstock, for example, by adding glass fibers in order to improve the mechanical properties of the printed object [74].

**Figure 5:**
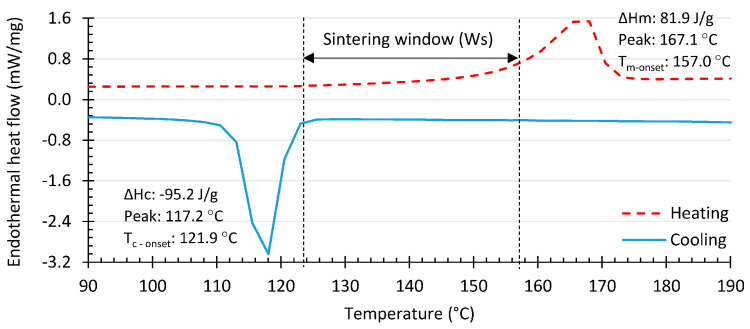
Differential scanning calorimetry thermograph of neat iPP (Advance3d materials) at a heating and cooling rate of 10 °C min${}^{-1}$. The sintering window is 35.1 °C, and the overall degree of crystallinity is 39.58%. Reproduced with permission [91], Copyright 2018, MDPI.

A second additive manufacturing technique used with iPP is SLS. An advantage of using iPP for SLS is the large sintering window of around 35 °C, as can be seen through the differential scanning calorimetry thermograph of Figure 5. In an SLS set-up, it is known that the temperatures can fluctuate, and due to the wide sintering window, the early crystallization can be delayed [91]. Nonetheless, temperature gradients can influence the type of crystals that are formed, as the degree of undercooling influences the crystallization kinetics of the different crystal morphologies [77]. Thus, a temperature gradient can give rise to different crystallization kinetics along the 3D-printed part. As a result, the mechanical properties can vary along the produced part.

Processing of iPP homopolymers and copolymers (CoPP) through SLS has been reported by Zhu et al. [92] and Tan et al. [93]. As expected, iPP homopolymer predominantly crystallized in the α-configuration. However, both works claim that using SLS for CoPP resulted in a substantial formation of γ-phase crystals. This is a special result, as the formation of γ-phase normally occurs under the condition of slow cooling and high pressure, with the application of high pressure being unlikely to occur during SLS [94,95,96]. Although the modulus and yield stress of the γ-phase are higher than for α-phase, the tensile strength of the γ-phase is lower [96]. Besides the evident appearance of the γ-phase, it was also found that the total crystallinity for iPP parts fabricated through SLS printing is higher compared to injection-molded counterparts. The increase in crystallinity can be explained through reorganization and crystal perfection as the 3D-printed part is kept at elevated temperatures during the SLS process. In addition, the cooling of the fabricated part to room temperature is performed in a more gradual manner compared to injection molding. Even though a higher tensile strength would be expected for specimens with a higher crystallinity, that is not the case, possibly due to the high porosity of the fabricated specimen [92].

In general, iPP can be processed through both SLS and FFF, but the main complexity is controlling the crystallization kinetics, temperature gradients, and porosity. An increase in adhesive fracture energy is challenging due to the early formation of crystals, either induced by flow or temperature, that prevent chain diffusion across the weld-line interfaces. The total crystallinity can be enhanced through post-printing thermal treatments, where the printed component is held at an elevated temperature between the crystallization and melting temperature [64].

### 3.2. Low- and High-Density Polyethylene

LDPE is branched polyethylene with a substantial number of long-chain branches. These branches do not fit in the polyethylene crystal lattice, resulting in a lower crystallinity compared to high-density polyethylene (HDPE) [97]. The lower crystallinity results in a lower density, hence the name “LDPE”. Because of its adjustable properties and low density, LDPE can be found in a wide range of applications, such as food packing and rigid containers [98]. Additive manufacturing of low-density polyethylene (LDPE) is considered to be possible but uninteresting due to its inferior mechanical properties. For this reason, only minimal research has been conducted on this matter.

Its low mechanical properties and poor adhesion pose difficulties toward a straightforward utilization of LDPE in additive manufacturing. The processing of LDPE through FFF has mainly been researched toward the formation of composites, where the polymer is reinforced with, for example, ceramic nano-particles or metal particles to improve the mechanical properties [99]. FFF processing of pure LDPE is explored in the work of Bedi et al. [100]. The main downsides of 3D printing with pure LDPE are reported to be a large amount of shrinkage and low mechanical properties. However, it was found that the reinforcement of LDPE, for instance, with alumina particles, was beneficial. Besides the mechanical properties, the crystallinity of the fabricated specimen was also increased, probably because the alumina particles act as nucleating agents during crystallization. SLS printing of LDPE has not been explored yet, probably due to a combination of reasons, such as bad adhesion, low crystallinity, low mechanical properties, and low melt viscosity, leading to dimensional instabilities during printing).

One of the most well-known polyolefins, and one of the leaders in commodity plastics, is high-density polyethylene (HDPE) due to its great potential for sustainability and recycling. HDPE is a linear polymer that can be processed by many common melt-processing means, for instance, through injection molding or extrusion [101]. HDPE is the second-most recycled plastic, and the ability to recycle HDPE in additive manufacturing, through FFF, has been demonstrated [102,103].

Schirmeister et al. [104] investigated the FFF of HDPE parts using a commercially available FFF printer. They found that they were able to match the mechanical properties of HDPE injection-molded specimens by carefully tuning the FFF process parameters. Extensive warping and porosity of the 3D-printed parts were avoided by using an appropriate build plate material and by increasing the extrusion rate during the printing process so that the formation of cavities is minimized and shrinkage is compensated for. A general problem with 3D printing HDPE that is encountered is the adhesion of the print object to the print surface. Schirmeister et al. [104] evaluated several print-surface materials and found that HDPE bonds best to a build plate made from HDPE but that detaching an HDPE FFF-printed sample from an HDPE build plate without damaging the build plate, or the printed object was virtually impossible, especially at higher nozzle temperatures. However, a good compromise was found by using a build plate made of poly(styrene-block-etheneco-butene-block-styrene) thermoplastic elastomer (SEBS, KRATON© FG1901 G), which exhibited good adhesion but still allowed for easy detachment of the printed object.

In the work of Mejia et al. [102], it is shown that HDPE can be recycled using FFF. The disadvantage of recycling HDPE is that the melt viscosity becomes higher, most probably due to the cross-linking that takes place during the processing cycles. This increasing viscosity makes the deposition of a filament with a constant diameter more difficult.

The processing of HDPE using SLS has been researched to a large extent in terms of blends, for example, with polyamide 12 (PA12), which is a standard material used in SLS. Through the use of PA12/HDPE blends, it has been shown that the sintering of HDPE particles is feasible [105,106]. Inter-diffusion of the macromolecular chains is impeded by the short sintering time [107,108]. The reptation time of HDPE, though depending strongly on molecular weight, is typically in the order of seconds, while the residence time of the material in the molten state during processing by SLS is mostly less than one second, thus limiting the amount of re-entanglement [62,109,110]. Moreover, porosity is a returning issue in SLS. It originates from the porous nature of polyethylene powder and the lack of consolidation of the powder upon printing, making SLS processing of HDPE even more challenging [29]. The porous naturem together with the relatively high crystallinity of HDPE, give rise to a large amount of shrinkage followed by warpage upon melting and subsequent cooling. In the work of Hoelzel et al. [111], the causes of the difference in the amount of warpage over each layer are investigated. In general, it was found that a higher scanning speed of the laser results in less warpage, which is due to a lower amount of powder melting, leading to less consolidation. When the scanning speed is lowered, more warpage occurs since the higher specific energy density applied to the scanned area penetrates the powder layer more. However, while more shrinkage and warpage are encountered, a larger specific energy density would improve the coalescence of the particles. The absorbance coefficient of HDPE can be improved by adding, for instance, a small amount of carbon black.

Another parameter that has to be considered is the flowability of the powder, which is typically low. In the work of Wencke et al. [112], the flowability was shown to be improved by coating the HDPE particles with nano-silica particles. This results in a printed object with a higher bulk density than expected; however, caking, i.e., the adhesion of particles to the sintered part around the printed object, is clearly present. Caking results from the melting of particles outside the melting region arising from irradiation scattering [93,112]. It is suggested that the addition of infrared-radiation-absorbing particles, such as carbon black or dyes, could decrease this scattering [112]. The mechanical properties of HDPEproduced samples through SLS have not been presently reported yet, possibly partly due to the inability to extract single, well-defined specimens due to caking.

### 3.3. Ultra-High Molecular Weight Polyethylene

Ultra-high molecular weight polyethylene (UHMWPE) is an engineering polymer used in a wide range of high-end applications ranging from medical implants to ballistic protection equipment due to its exceptional properties, such as high impact strength, high abrasive resistance, high chemical resistance, and biocompatibility. However, the main reason for the exceptional mechanical properties, namely the ultra-long macromolecular chains, result in ultra-high melt viscosity, rendering melt processing of UHMWPE a tedious process. For this reason, UHMWPE is regarded as an intractable polymer that cannot be processed by common melt-processing techniques used for polymers, such as injection molding and melt extrusion. UHMWPE is typically sold as as-polymerized reactor powder and processed through compression molding or ram extrusion into plates and rods, followed by machining to obtain the desired parts. Both processes severely hamper the design flexibility of acquired parts and have low throughput. Hence, the fabrication of UHMWPE parts through additive manufacturing would significantly widen the processing pathways for UHMWPE and the design flexibility necessary for specific applications, such as, for example, personalized medical implants.

**Figure 6:**
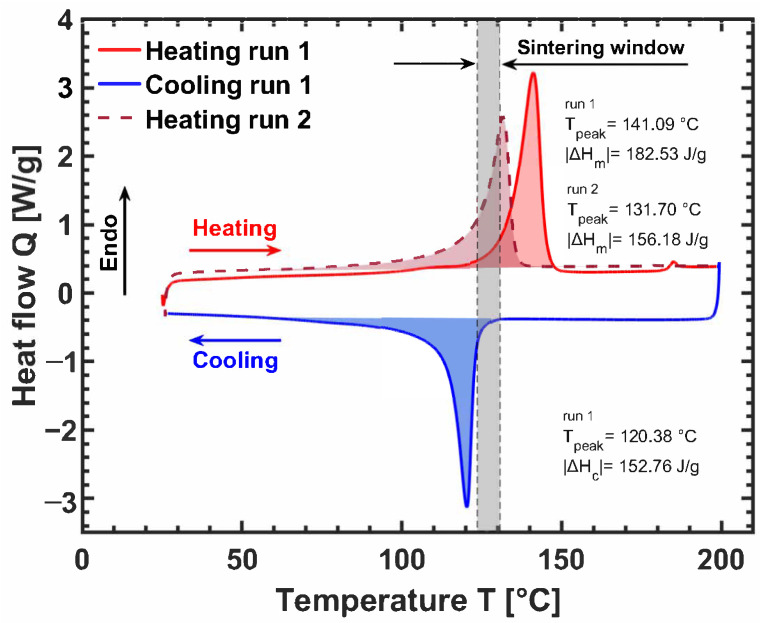
Differential scanning calorimetry thermograph of reactor powder UHMWPE (GUR4120, molecular weight of 4.5 $\times \;10^{6}$g mol${}^{-1}$) at a heating and cooling rate of 10 °C min${}^{-1}$. The red solid and red dashed lines correspond to the melting of the as-polymerized and melt-crystallized powder, respectively. The sintering window is approximately 7 °C.

Due to the limitation of melt extrusion, processing through FFF of pure UHMWPE is not feasible. However, there have been several attempts to process UHMWPE through SLS. The sintering window for UHMWPE can be seen in Figure 6, where a differentialscanning calorimetry thermograph of a standard UHMWPE grade (GUR4120 supplied from Celanese) is depicted. The experiments were conducted on a Discovery 2500 differential scanning calorimeter (TA Instruments), with a heating/cooling rate of 10 °C min${}^{-1}$, under nitrogen flow. UHMWPE is known to depict superheating, i.e., the melting peak temperature of the as-polymerized powder is higher (141 °C) than that of subsequent melting runs (∼132 °C) [113,114,115]. Since the powder used for SLS is reactor powder, and the melt viscosity of UHMWPE is so high that it is practically intractable, the high melting peak temperature of about 141 °C is used for estimating the temperature window for sintering.

The works of Goodridge et al. [116], Khalil et al. [117], Song et al. [118], and Ullsperger et al. [119] suggest that there is considerable room for improvement in the SLS printing of UHMWPE. A common denominative in the aforementioned works is a significant decrease in mechanical properties, such as tensile strength and elongation at break, of the printed specimen compared to the specimen fabricated through compression molding. A typical value for the tensile strength of UHMWPE processed through compression molding is about 50 MPa, while the values found in the literature for specimens fabricated through SLS range between 5 and 14 MPa [117,118,119]. This could be due to the low level of re-entanglement between the sintered polymer chains. Re-entanglement, through chain diffusion, is essential for the development of good mechanical properties [44,46,120,121,122,123]. As mentioned earlier, re-entanglement of the macromolecular chains in polymer melts occurs through reptation of the polymer chains with the characteristic reptation time scaling with the $M_{w}$. Selfdiffusion, i.e., reptation, of the polymer chains is a slow process for UHMWPE due to its ultra-high molecular weight, which is typically higher than 3,000,000 g mol${}^{-1}$, resulting in a high amount of steric hindrance between the polymer chains. This steric hindrance is often expressed as the number of entanglements per chain. Given a molecular weight between entanglements for polyethylene of 1250 g/mol, this means that UHMWPE has more than 2400 entanglements per chain, which is high compared to melt-processable grades of polyethylene that typically have about 100–200 entanglements per chain.

The ultra-long polymer chains of UHMWPE result in the longest relaxation time in the melt being in the order of hours, which is orders of magnitude larger than the residence time in the melt when processed through SLS [124]. However, it should be noted that full re-entanglement across the interface upon welding of two UHMWPE surfaces might not be necessary to obtain a good adhesion at room temperature. Xue et al. [46] reported that good adhesion between UHMWPE surfaces, expressed as the strength of a weld as determined by T-peel testing at room temperature, can be achieved by co-crystallization of the macromolecular chains across the interface. However, the effect of this mechanism on 3D-printed specimens has not been observed yet.

**Figure 7:**
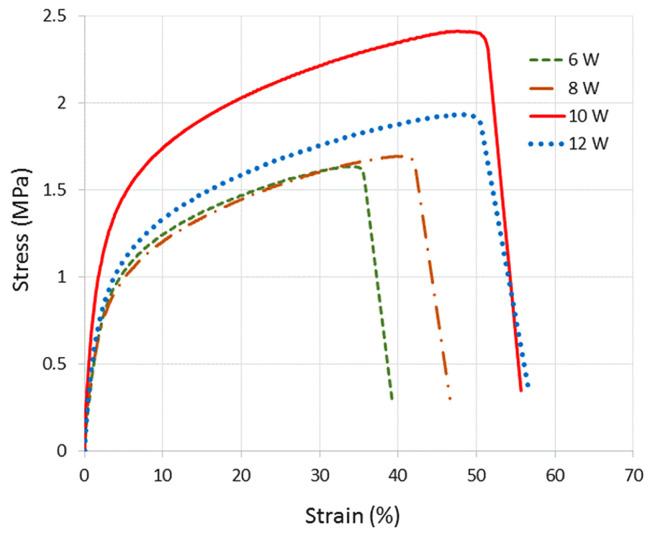
Engineering stress against engineering strain for different laser-sintered UHMWPE samples with laser powers of 6, 8, 10, and 12 W, respectively. Each profile constitutes the average of five samples tested under similar conditions. Reproduced with permission [117], Copyright 2016, EDP Sciences.

The first-reported example of UHMWPE sintering into specimens with sufficient mechanical strength that could be removed from the powder bed is from Goodridge et al. [116], where the heating of the powder bed to a temperature close, but below, the melting temperature was implemented in order to promote melting. Due to the brittle nature of the specimen, the flexural strength, instead of the more commonly evaluated tensile strength, was measured for these samples, with the average value being only about 0.52 MPa. Differential scanning calorimetry experiments of these sintered parts showed that the powder was only partially molten during sintering, as still a large fraction of the original, hightemperature, melting peak of nascent UHMWPE powder could be observed. In general, incomplete fusion of particles appears to be an important factor that limits the ultimate tensile strength and appears to be directly related to the laser power used to sinter the powder during SLS printing. In Figure 7, the stress-strain curves at room temperature for UHMWPE samples produced with different amounts of applied laser power are shown. For the parameters used in this study, this graph suggests an optimum laser power of 10 W. However, the influence of wavelength, hatch spacing, and laser speed must also be taken into account [125].

As mentioned before, the specific energy density is related to the amount of energy provided per unit area. The specific energy density $E_{s}$ is defined as follows [116]:

(1) $$E_{s}=\frac{P_{\mathrm{av}}}{v_{l}\cdot h},$$

where $P_{\mathrm{av}}$ is the average laser power, $v_{l}$ is the speed of the laser, and *h* is the hatch spacing. Degradation of the polymer is the result of the specific energy density being too high, such that thermal degradation occurs, leading to chain scission and decomposition of the macromolecular chains due to irradiation and buildup of superfluous heat [119,125]. Nonetheless, the incident energy is not to be mistaken with the absorbed energy. The processing of UHMWPE through SLS is hindered by the fact that the absorption coefficient is very low, about 0.01 cm${}^{-1}$ at a wavelength of 1064 nm [126]. As a result, the excessive heat gives rise to a decrease in resolution, increases warpage, and leads to oxidative degradation processes [38].

**Figure 8:**
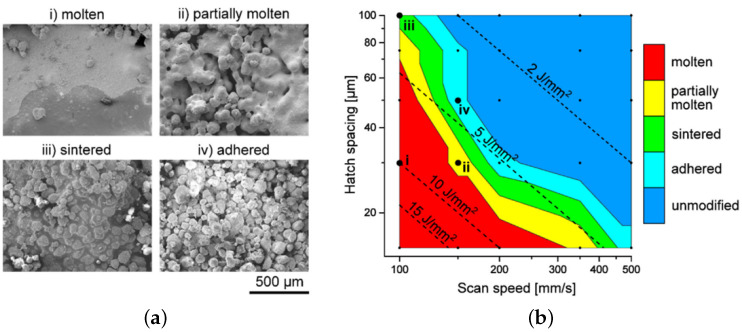
(**a**) Scanning electron microscopy micrographs of four different UHMWPE samples made via SLS printing. (**b**) Characterization of the consolidation of UHMWPE via SLS printing, where the hatch spacing is shown against the scan speed of the laser. Here, a direct correlation between the characterization and the specific energy density is shown. Reproduced with permission [119], Copyright 2021, Elsevier.

In the work of Ullsperger et al. [119], a characterization of the consolidation regime related to the specific energy density has been performed, as shown in Figure 8. Four different consolidation regimes were identified, ranging from weakly sintered particles to complete melting. The optimal specific energy density was determined to be between 6 and 8 J mm${}^{-2}$.

Their work highlights the inherent difficulty of processing UHMWPE through SLS [119]. It was found that the narrow sintering window is not only dependent on temperature but also on the laser speed, hatch spacing, and layer height. Furthermore, tensile bars were fabricated at the optimal sintering conditions, where a well-sintered layer is achieved. However, the mechanical properties still remain low compared to the compression molded specimen, possibly due to embedded pores and fusion defects at the layer boundaries. In addition, the porous nature of the UHMWPE reactor powder causes additional shrinkage when achieving a well-sintered layer.

## 4. Outlook

The additive manufacturing of polyolefins is still a difficult process, and for polyolefins to be added to the standard palette of materials used in additive manufacturing, significant improvements are necessary. The development of the mechanical properties of printed parts is impeded by the slow formation of entanglements across an interface. Regarding FFF, the shape of the filament is prone to generating porous 3D-printed samples. The residence time in the melt can be increased by lowering the thermal gradient between the nozzle and bed temperature. A further benefit of keeping the deposited filaments above the melting point for a longer time is to promote the increased mobility of the polymer chains and subsequent re-entanglement toward increased mechanical properties. However, the residence time in the melt is naturally limited by the amount of flow, which relates to the viscosity of the given polymer.

Apart from the increase in design flexibility, a further benefit of processing polyolefins through additive manufacturing is the possibility of plastic recycling. It is claimed in the work of Vidakis et al. [127] that for iPP, the mechanical properties are not severely decreased upon recycling via FFF. Nevertheless, thermal degradation of the polymer is likely to occur after each processing cycle, leading to reduced molecular weights, which will decrease the mechanical performance. However, re-using powder used in selective laser sintering is preferred since the unused powder that is found in the support bed is not degraded that much as it remains unsintered. Nevertheless, since the whole chamber is being heated, thermal degradation of the recycled powder can still occur after several heat cycles. The current limitation of processing polyolefins through SLS is that both the powder fusion and flowability of powder particles are poor. The flowability in SLS is mainly dependent on the bulk packing density. A lower bulk packing density is preferred for good flowability but increases porosity, especially for polyolefins with high-viscous melts, such as UHMWPE. A possible solution, which is also used in non-commercial SLS set-ups, is the use of a roller for compacting. The combination of a slider for the distribution of the powder and subsequent compacting using a roller could result in a higher packing density in the build compartment of an SLS printer while retaining good flowability during the deposition of each new powder layer. Denser packing before sintering will naturally improve the fusion of two particles, as the contact between particles, both in the same and in adjacent layers, is improved, and the overall porosity is decreased. Another complicating factor when processing polyethylene through SLS is its low absorbance coefficient. This could be improved by the addition of a coloring agent to color the powder so that the wavelength of the laser can be absorbed by it. Lastly, for the case of UHMWPE processed through SLS, good adhesion between the powder particles could be achieved by using disentangled UHWMPE powder that has been shown to rapidly re-entangle upon melting, through a mechanism that deviates from slow reptation kinetics [128].

## Data Availability

Not applicable.
